# Towards a multi-antigen multi-stage malaria vaccine

**DOI:** 10.1186/1475-2875-13-S1-O31

**Published:** 2014-09-22

**Authors:** Adrian VS Hill, Sumi Biswas, Simon Draper, Thomas Rampling, Arturo Reyes-Sandoval

**Affiliations:** 1 University of Oxford, UK

## 

A highly effective malaria vaccine is a major goal of global health research and will likely require a multi-stage product. Oxford researchers are developing the concept of a highly effective multi-stage *P. falciparum* vaccine to the point of proof-of-concept phase II testing in Europe, prior to trials in malaria-endemic areas.

Remarkable recent advances in vaccine design for all four stages of the *P. falciparum* parasite’s life-cycle allow testing of a multi-stage multi-component vaccine for the first time, with strong chances of success. These advances are i) the availability of a new vectored prime-boost vaccination regime based on the chimpanzee adenovirus technology that has been found to induce exceptionally potent CD8^+^ T cell responses and high titre antibodies against multiple malaria antigens; ii) the development of an improved virus-like particle (VLP) version of the leading partially protective RTS,S sporozoite vaccine candidate, termed R21, that lacks the excess of HBsAg in RTS,S; iii) the identification, using a vector technology screen, of the blood-stage antigen RH5 as the first antigen to induce potent strain-transcending neutralization of blood-stage parasites in *in vitro* growth inhibition assays; and iv) the demonstration that antibodies against a mosquito-stage antigens that induce 100% transmission blocking against field isolates of *P. falciparum* in Africa are inducible by a new nanoparticle vaccine candidate.

In parallel similar approaches using vectors and VLPs are underway to target the pre-erythrocytic stages of *P. vivax*, including the hypnozoite, and a phase I trial of the vivax blood-stage vaccine candidate, PvRII, is nearing completion.

We are aiming to undertake phase I/II clinical trials to assess the *P. falciparum* pre-erythrocytic, blood-stage and mosquito-stage components individually, and then together, using state-of-the art immunomonitoring, key functional assays of vaccine-induced immunogenicity, and sporozoite and blood-stage parasite challenges to measure efficacy prior to field testing. An update on this programme will be presented. A viral vectored prime-boost regime has recently shown high efficacy against malaria infection in East Africa and the first combination trial of RTS,S/AS01 with these vectors has been completed.

The prospects for achieving high efficacy with such combination approaches now appear very good.

